# Lupus miliaris disseminatus faciei with extra-facial involvement in a 6-year-old Japanese girl^⋆^

**DOI:** 10.1016/j.abd.2022.11.009

**Published:** 2024-04-23

**Authors:** Misaki Kusano, Maki Takada, Natsuko Matsumura, Toshiyuki Yamamoto

**Affiliations:** Department of Dermatology, Fukushima Medical University, Fukushima, Japan

Dear Editor,

Lupus miliaris disseminatus faciei (LMDF) predominantly occurs at 20–30 years of age and is rarely seen in children. We report a pediatric case of LMDF affecting the face and the labia majora.

A 6-year-old Japanese girl was referred to our department, complaining of a 5-month history of pruritic papular eruptions on the face. She had been treated with oral antiallergic drugs and topical corticosteroids, but without effects. Physical examination showed numerous 1‒2 mm dome-shaped small reddish papulonodules around the mouth and lower eyelids (Fig. 1 A and B). In addition, reddish papules were scattered in the labia majora (Fig. 2). A skin biopsy was carried out from papular eruptions on the right jaw. Histological examination revealed dermal epithelioid cell granulomas without caseous necrosis (Fig. 3A). Magnified epithelioid cell granuloma images showed multinucleated giant cells in the dermis (Fig. 3B), as well as inflammatory lymphocytic and histiocytic infiltration around the granulomas and hair follicles area. Immunostaining was positive for CD68 and CD163 antigens (Fig. 3 C and D). A tuberculin test was negative. Treatment with oral administration of clarithromycin showed favorable effects on the vulvar lesions after 5 months and the facial lesions after 9 months.

**Figure 1:**
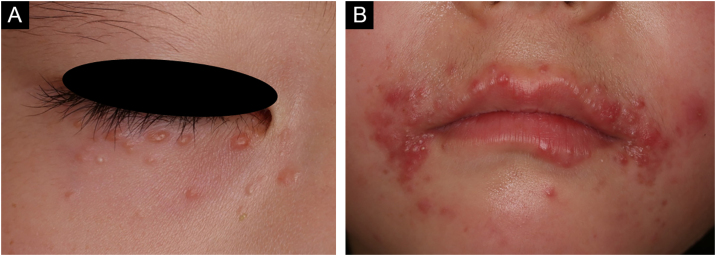
Numerous small erythematous papules were located on the lower eyelid (A) and around the mouth (B).

**Figure 2:**
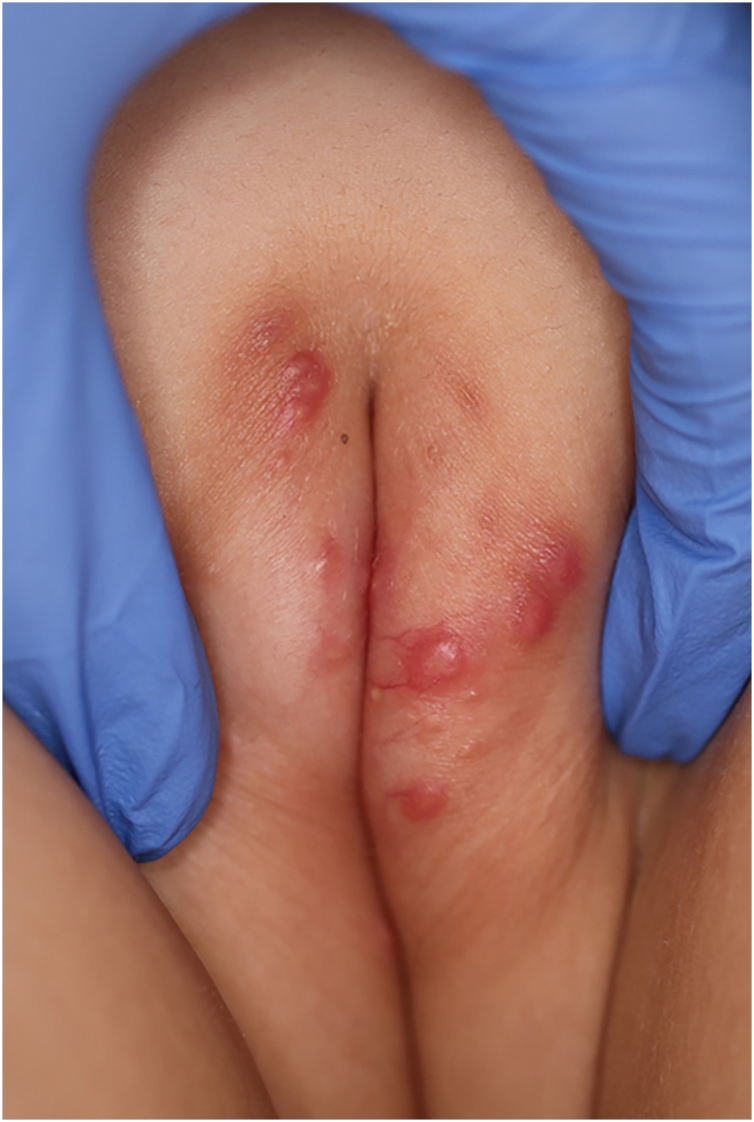
Reddish papules were scattered in the labia majora.

**Figure 3:**
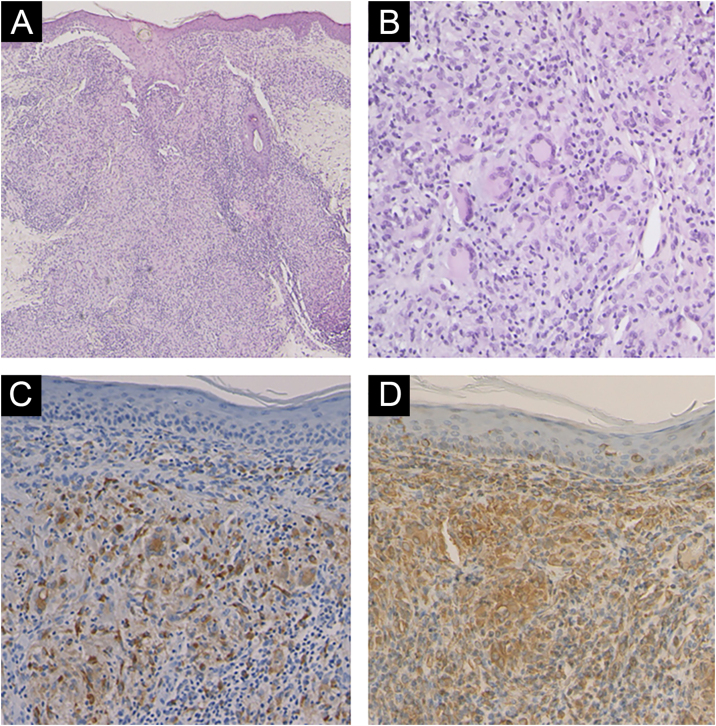
Histopathological features showing epithelioid cell granulomas within the dermis (A). Higher magnification reveals epithelioid cell granulomas containing multinucleated giant cells (B). Immunohistochemistry revealed positive findings for CD68 (C) and CD163 (D).

The present case developed multiple palpebral and perioral papulonodular lesions, which are the frequently involved sites of LMDF. In addition, vulvar involvement was observed. The patient did not have any organ symptoms suggestive of juvenile-onset sarcoidosis. Granulomatous rosacea was excluded, because neither facial erythema nor telangiectasia was observed, and the patient denied flushing.

Clinical and pathological features of pediatric LMDF are slightly different from adult LMDF, such as i) Papules concentrated around the mouth, on the nasolabial fold, and on the lower eyelids, ii) Small papule size, iii) Few pustules and scarring, iv) Redness around the mouth, v) Few caseous necrosis within epithelioid granulomas, and vi) Short clinical course.^1^ On the other hand, Childhood Granulomatous Periorificial Dermatitis (CGPD) was reported as a disease in which yellow-brown papular eruptions limited to the perioral, perinasal, and periocular regions that histopathologically show epithelioid cell granulomas around hair follicles.^2^ The features of LMDF in childhood are highly similar to those of CGPD, and differentiation of both disorders is challenging and possibly both disorders are the same entity.^1^

There are several cases of LMDF with extra-facial involvement in sites such as the neck, axilla, groin, and extremities. Genital regions are also affected, and to our knowledge, there are only four reported cases of this involvement in childhood LMDF (1 case) and CGPD (3 cases) (Table 1).^3^, ^4^ The age of onset was 6–9 years, and all cases were girls. They were successfully treated with oral minocycline, topical non-steroidal anti-inflammatory drugs, oral and topical erythromycin, topical metronidazole, topical tacrolimus, and topical metronidazole.^4^, ^5^ Treatment response is better in pediatric LMDF than in adult cases. In pediatric cases of LMDF, genital areas should be examined in detail.

**Table 1:** Cases of vulvar involvement in childhood Lupus miliaris disseminatus faciei and childhood granulomatous periorificial dermatitis.

Author and year of publication	Disease	Age in years	Gender	Clinical features	Treatment	Treatment duration
Wataeda et al. (1990)	LMDF in children	9	Female	Face, labia majora	Oral minocycline, topical non-steroidal anti-inflammatory drug	3 mo and a half
Andry et al. (1995)	CGPD	Unknown	Female	Face, perivulvar	Unknown	Unknown
Amy et al. (2002)	CGPD	6	Female	Face, labia majora	Oral and topical erythromycin	2 mo
Amy et al. (2002)	CGPD	8	Female	Face, arms, abdomen, labia majora	Oral erythromycin, topical metronidazole	Unknown

## Financial support

None declared.

## Authors’ contributions

Misaki Kusano: Approval of the final version of the manuscript; critical literature review; data collection; analysis and interpretation; study conception and planning; management of studied cases; manuscript critical review; preparation and writing of the manuscript.

Maki Takada: Approval of the final version of the manuscript; critical literature review; manuscript critical review; preparation and writing of the manuscript.

Natsuko Matsumura: Approval of the final version of the manuscript; critical literature review; manuscript critical review; preparation and writing of the manuscript.

Toshiyuki Yamamoto: Approval of the final version of the manuscript; critical literature review; data collection; analysis and interpretation; study conception and planning; manuscript critical review; preparation and writing of the manuscript.

## Conflicts of interest

None declared.
